# ﻿Comparative mitogenomics and phylogenetic analyses of the genus *Menida* (Hemiptera, Heteroptera, Pentatomidae)

**DOI:** 10.3897/zookeys.1138.95626

**Published:** 2023-01-05

**Authors:** Xiaofei Ding, Chao Chen, Jiufeng Wei, Xiaoyun Gao, Hufang Zhang, Qing Zhao

**Affiliations:** 1 College of Plant Protection, Shanxi Agricultural University, Taigu 030801, Shanxi, China Shanxi Agricultural University Taigu China; 2 Department of Biology, Xinzhou Teachers University, Xinzhou 034000, Shanxi, China Xinzhou Teachers University Xinzhou China

**Keywords:** *Menida*, mitochondrial genomesm Pentatominae, phylogenetic relationship

## Abstract

In order to explore the genetic diversity and phylogenetic relationship of the genus *Menida* Motschulsky, 1861 and reveal the molecular evolution of the family Pentatomidae, subfamily Pentatominae, complete mitochondrial genomes of three species of *Menida* were sequenced, and the phylogenetic relationships of tribes within the subfamily Pentatominae were studied based on these results. The mitochondrial genomes of *Menidamusiva* (Jakovlev, 1876), *M.lata* Yang, 1934, and *M.metallica* Hsiao & Cheng, 1977 were 16,663 bp, 16,463 bp, and 16,418 bp, respectively, encoding 37 genes and including 13 protein-coding genes (PCGs), two rRNA genes, 22 tRNA genes, and a control region. The mitochondrial genome characteristics of *Menida* were compared and analyzed, and the phylogenetic tree of the Pentatominae was constructed based on the mitochondrial genome datasets using Bayesian inference (BI) and maximum likelihood (MI) methods. The results showed that gene arrangements, nucleotide composition, codon preference, gene overlaps, and RNA secondary structures were highly conserved within the *Menida* and had more similar characteristics in Pentatominae. The phylogenetic analysis shows a highly consistent topological structure based on BI and ML methods, which supported that the genus *Menida* belongs to the Pentatominae and is closely related to Hoplistoderini. The examined East Asian species of *Menida* form a monophyletic group with the internal relationships: (*M.musiva* + (*M.lata* + (*M.violacea* + *M.metallica*))). In addition, these results support the monophyly of Eysarcorini and Strachiini. *Placosternum* and Cappaeini are stable sister groups in the evolutionary branch of Pentatominae. The results of this study enrich the mitochondrial genome databases of Pentatominae and have significance for further elucidation of the phylogenetic relationships within the Pentatominae.

## ﻿Introduction

Mitochondrial genomes are one of the most widely used molecular markers in evolutionary studies due to their small size, stable genetic composition, relatively conserved gene sequence, rapid rate of evolution, and relatively complete molecular information (Wolstenholme 1992; Chen et al. 2020a). In recent years, with the development of sequencing technology, more and more insect mitochondrial genomes have been sequenced, covering almost all insect orders. A typical insect mitochondrial genome comprises circular double-stranded molecules 15–20 kb in size that usually code for 37 genes: 13 protein-coding genes (PCGs), two ribosomal RNA genes (rRNAs), 22 transfer RNA genes (tRNAs), and a control region (also known as AT-rich region) (Boore 1999). The structure of insect mitochondrial genomes is compact, the overlap region and spacing region of adjacent genes are very short, and there are no introns (Zink 2005). Insect mitochondrial genomes are widely used in molecular evolution, phylogenetic and population genetic structure analyses, and biogeographic studies (Simon and Hadrys 2013; Yuan and Guo 2016; Wang et al. 2017; Wang et al. 2020; Zheng et al. 2021).

Pentatominae is the largest subfamily of Pentatomidae, which is composed of at least 3484 species belonging to 660 genera in 43 tribes (Rider et al. 2018). Species feed on the liquid flowing in the host plant’s vegetative organs using piercing-sucking mouthparts; they suck up nutrients in the host plant and make it shrink and dry. They cause great losses to crops, vegetables, fruit trees, and forests, and, as such, are important agricultural pests (Mi et al. 2020). The lack of unique diagnostic characteristics hampers the identification of this subfamily, making it difficult to construct criteria for practical and reliable classification. Most previous studies have focused on the high-level relationships within Pentatomoidea, while the phylogenetic relationships of tribes within Pentatominae remain controversial. Liu et al. (2019) reconstructed the phylogeny of Pentatomomorpha based on the PCGrRNA dataset under the Bayesian site-heterogeneous mixture model, and they examined the evolutionary history of the group through a fossil-calibrated divergence dating analysis, confirming the monophyly of Pentatomoidea and its sister relationship with Eutrichophora. Ye et al. (2022) also presented a phylogenetic analysis. Yuan et al. (2015) constructed the phylogenetic tree of Pentatomoidea based on mitochondrial genome data, which strongly supported the monophyly of Pentatomoidea. The data produced by Zhao et al. (2019b) strongly supported *Eurydema* Laporte, 1833 within the tribe Strachiini and as a sister group with *Nezaraviridula* (Linnaeus, 1758). Genevcius et al. (2021) confirmed that the currently recognized Neotropical tribe Chlorocorini is not monophyletic based on DNA and morphological data. Roca-Cusachs et al. (2022) rejected the currently accepted monophyletic nature of Pentatomidae, confirming that Serbaninae are a sister lineage of all remaining Pentatomidae, rather than members of Phloeidae as previously assumed. Li et al. (2021) studied the phylogenetic relationships among the groups of Pentatominae and supported the placement of *Eysarcoris* Hahn, 1834 and *Carbula* Stål, 1864 in Eysarcorini.

The genus *Menida* Motschulsky, 1861 is distributed worldwide, but most species are distributed in Afrotropical and Oriental regions (Li 2015). Species of the genus *Menida* pierce the surface of the host plant and sucks the liquid in the plant using piercing-sucking mouthparts. This destroys the plant’s tissue and causes loss of water, thus causing the plant to suffer from such diseases as withering spot and decay. Examples are *Menidaversicolor* (Gmelin, 1790) feeding on and damaging rice and *Menidapinicola* Zheng & Liu, 1987 feeding on and damaging pine trees. The body shape of *Menida* species is oval, and the surface is often with a metallic luster and color spots. However, the body color is variable and some species have a large range of variation (Li 2015), which can cause difficulties in identifications. Most of the research on the genus has focused on morphology or biology and less on the mitochondrial genome (Dai and Zheng 2005; Li et al. 2015; Markova et al. 2020).

In this study, we newly sequenced the complete mitochondrial genomes of three species of *Menida* based on high-throughput sequencing, analyzed the characteristics of the mitochondrial genome in detail and drew the secondary structure of RNA. By comparing and analyzing the characteristics of mitochondrial genome sizes, nucleotide composition, codon preference, RNA structure, and evolutionary rates among *Menida* species, we explore the phylogenetic position of *Menida* in Pentatominae, as well as the relationship of tribes within the subfamily Pentatominae. The new data will provide a reference for the phylogenetic analysis and identification of Pentatomidae.

## ﻿Materials and methods

### ﻿Sample collection and DNA extraction

Adult specimens of *Menidamusiva* (Jakovlev, 1876) were collected from Gaoleshan National Nature Reserve (32°39.90'N, 113°37.37'E), Tongbai County, Nanyang City, Henan Province, China, in August 2019. Adult specimens of *M.lata* Yang, 1934 were collected from Buddhist College of Tongbo County (32°21'N, 113°23'E), Nanyang City, Henan Province, China, in August 2019. Adult specimens of *M.metallica* Hsiao & Cheng, 1977 were collected from Wuli Village (30°52'N, 103°35'E), Qingchengshan Town, Dujiangyan City, Sichuan Province, China, in September 2020. All samples were immediately placed in absolute ethanol and stored in a freezer at −20 °C until DNA extraction. The total DNA was extracted from thoracic tissue using the HiPure Universal DNA Kit (Jisi Huiyuan biotechnology, Nanjing, China).

### ﻿Sequencing, assembly, annotation, and bioinformatics analyses

The complete mitochondrial genomes of the three species were sequenced on Illumina Novaseq 6000 Sequencing System with a read length of PE150. Fastp (Chen et al. 2018) software was used to filter the original data and remove the joint sequences and low-quality reads to obtain high-quality, clean data. Three mitochondrial genomes were assembled using SPAdes v. 3.10.1 (Bankevich et al. 2012), and the assembly of the genomes did not depend on the reference genome. After assembly, the complete mitogenomes were manually annotated using Geneious v. 11.0 (Kearse et al. 2012) software. A reference sequence of *M.violacea* for annotation was obtained from the basic local alignment search tool (BLAST) in the NCBI database. PCGs were identified by open reading frame (ORF) Finder (http://www.ncbi.nlm.nih.gov/gorf/gorf.html) implemented through the NCBI website using invertebrate mitochondrial genetic codes. The position and structure of 22 tRNAs were predicted using the MITOS Web Server (http://mitos.bioinf.uni-leipzig.de/index.py/) (Bernt et al. 2013). The exact locations of rRNA adjacent genes and the control regions were determined by confirming the boundary between them. In addition, tandem repeats of the control region were identified with the Tandem Repeats Finder server (http://tandem.bu.edu/trf/trf.html) (Benson 1999).

The circular maps of mitogenomes were produced by the CGView Server (Grant and Stothard 2008). Nucleotide composition and codon usage were analyzed with MEGA v. 11 (Tamura et al. 2021). To investigate the evolutionary patterns among the mitochondrial PCGs in Pentatominae species, DnaSP5 software (Librado and Rozas 2009) was used to count the non-synonymous substitutions (Ka) and synonymous substitutions (Ks) of 13 PCGs of Pentatominae, and to calculate the Ka/Ks values. The skew of the nucleotide composition was calculated with the formulas: AT-skew = (A − T) / (A + T) and GC-skew = (G − C) / (G + C) (Perna and Kocher 1995).

### ﻿Phylogenetic analysis

We selected three newly sequenced species of *Menida* and 37 available mitogenomes of related taxa (including all available Pentatominae sequences and two Acanthosomatidae sequences as outgroups) from GenBank to determine the phylogenetic status of *Menida* and to discuss the phylogenetic relationships of tribes within the subfamily Pentatominae (Table 1). The phylogenetic relationships were reconstructed based on two datasets: (1) 13 PCGs + 2 rRNAs (PR) and (2) 13 PCGs + 2 rRNAs + 22 tRNAs (PRT). The two data sets represent relatively complete genetic evolution information of mitochondrial genomes.

**Table 1.** List of sequences used to reconstruct the phylogenetic relationships within Pentatominae.

Family	Subfamily	Tribe	Species	GenBank number	Reference
Pentatomidae	Pentatominae	Antestiini	*Anaxilausmusgravei*	MW679031	Unpublished
		Antestiini	*Plautiacrossota*	NC_057080	(Wang et al. 2019)
		Antestiini	*Plautiafimbriata*	NC_042813	(Liu et al. 2019)
		Antestiini	*Plautialushanica*	NC_058973	(Xu et al. 2021)
		Cappaeini	*Halyomorphahalys*	NC_013272	(Lee et al. 2009)
		Carpocorini	*Dolycorisbaccarum*	NC_020373	(Zhang et al. 2013)
		Catacanthini	*Catacanthusincarnatus*	NC_042804	(Liu et al. 2019)
		Caystrini	*Caystrusobscurus*	NC_042805	(Liu et al. 2019)
		Caystrini	*Hippotiscusdorsalis*	NC_058969	(Xu et al. 2021)
		Eysarcorini	*Carbulasinica*	NC_037741	(Jiang 2017)
		Eysarcorini	*Eysarcorisaeneus*	MK841489	(Zhao et al. 2019a)
		Eysarcorini	*Eysarcorisannamita*	MW852483	(Li et al. 2021)
		Eysarcorini	*Stagonomusgibbosus*	MW846868	(Li et al. 2021)
		Eysarcorini	*Eysarcorisguttigerus*	NC_047222	(Chen et al. 2020b)
		Eysarcorini	*Eysarcorismontivagus*	MW846867	(Li et al. 2021)
		Eysarcorini	*Eysarcorisrosaceus*	MT165687	(Li et al. 2021)
		Halyini	*Dalpadacinctipes*	NC_058967	(Xu et al. 2021)
		Halyini	*Erthesinafullo*	NC_042202	(Ji et al. 2019)
		Hoplistoderini	*Hoplistoderaincisa*	NC_042799	(Liu et al. 2019)
		Menidini	*Menidamusiva*	OP066239	This study
		Menidini	*Menidametallica*	OP066240	This study
		Menidini	*Menidalata*	OP066241	This study
		Menidini	*Menidaviolacea*	NC_042818	(Liu et al. 2019)
		Nezarini	*Glauciasdorsalis*	NC_058968	(Xu et al. 2021)
		Nezarini	*Nezaraviridula*	NC_011755	(Hua et al. 2008)
		Nezarini	*Palomenaviridissima*	NC_050166	(Chen et al. 2021)
		Pentatomini	*Neojurtinatypica*	NC_058971	(Xu et al. 2021)
		Pentatomini	*Pentatomametallifera*	NC_058972	(Xu et al. 2021)
		Pentatomini	*Pentatomarufipes*	MT861131	(Zhao et al 2021)
		Pentatomini	*Pentatomasemiannulata*	NC_053653	(Wang et al. 2021)
		Pentatomini	*Placosternumurus*	NC_042812	(Liu et al. 2019)
		Sephelini	*Brachymnatenuis*	NC_042802	(Liu et al. 2019)
		Strachiini	*Eurydemadominulus*	NC_044762	(Zhao et al. 2019b)
		Strachiini	*Eurydemagebleri*	NC_027489	(Yuan et al. 2015)
		Strachiini	*Eurydemaliturifera*	NC_044763	(Zhao et al. 2019b)
		Strachiini	*Eurydemamaracandica*	NC_037042	(Zhao et al. 2017)
		Strachiini	*Eurydemaoleracea*	NC_044764	(Zhao et al. 2019b)
		Strachiini	*Eurydemaqinlingensis*	NC_044765	(Zhao et al. 2019b)
Acanthosomatidae	Acanthosomatinae		*Anaxandrataurina*	NC_042801	(Liu et al. 2019)
			*Sastragalaesakii*	NC_058975	(Xu et al. 2021)

The nucleic acid sequences of the PCGs and RNA genes were extracted using Geneious v. 11.0 and aligned using the MUSCLE strategy in MEGA v. 11. Multiple sequences for each species were then connected using SequenceMatrix v. 1.7.8 (Vaidya et al. 2011), protein-coding genes were optimized using MACSE (Ranwez et al. 2011), ambiguous loci were deleted using Gblocks (Talavera and Castresana 2007), and converted into Nexus and Phylip formats in Mesquite v. 3.7 (Maddison 2008). To determine the best model for partitioning, four datasets were analyzed using PartitionFinder v. 2.1.1 (Lanfear et al. 2017). The maximum likelihood (ML) and Bayesian inference (BI) methods were used for phylogenetic analysis based on two datasets. The ML trees were constructed by IQ-TREE v. 2.2.0 (Minh et al. 2020), and the support value for each node was evaluated by the standard bootstrap (BS) algorithm, which was tested 500,000 times. The Bayesian inference (BI) method was used for phylogenetic analysis based on four datasets. The BI tree was constructed by MrBayes v. 3.2.7 (Ronquist et al. 2012). Two independent runs were run for 10 million generations, and samples were taken every 1000 generations. Four independent Markov Chains (including three heated chains and a cold chain) were run. A consensus tree was obtained from all the trees after the initial 25% of trees from each MCMC run were discarded as burn-in, with the chain convergence assumed after the average standard deviation of split frequencies fell below 0.01.

## ﻿Results

### ﻿Mitochondrial genomic structure

**Figure 1.**
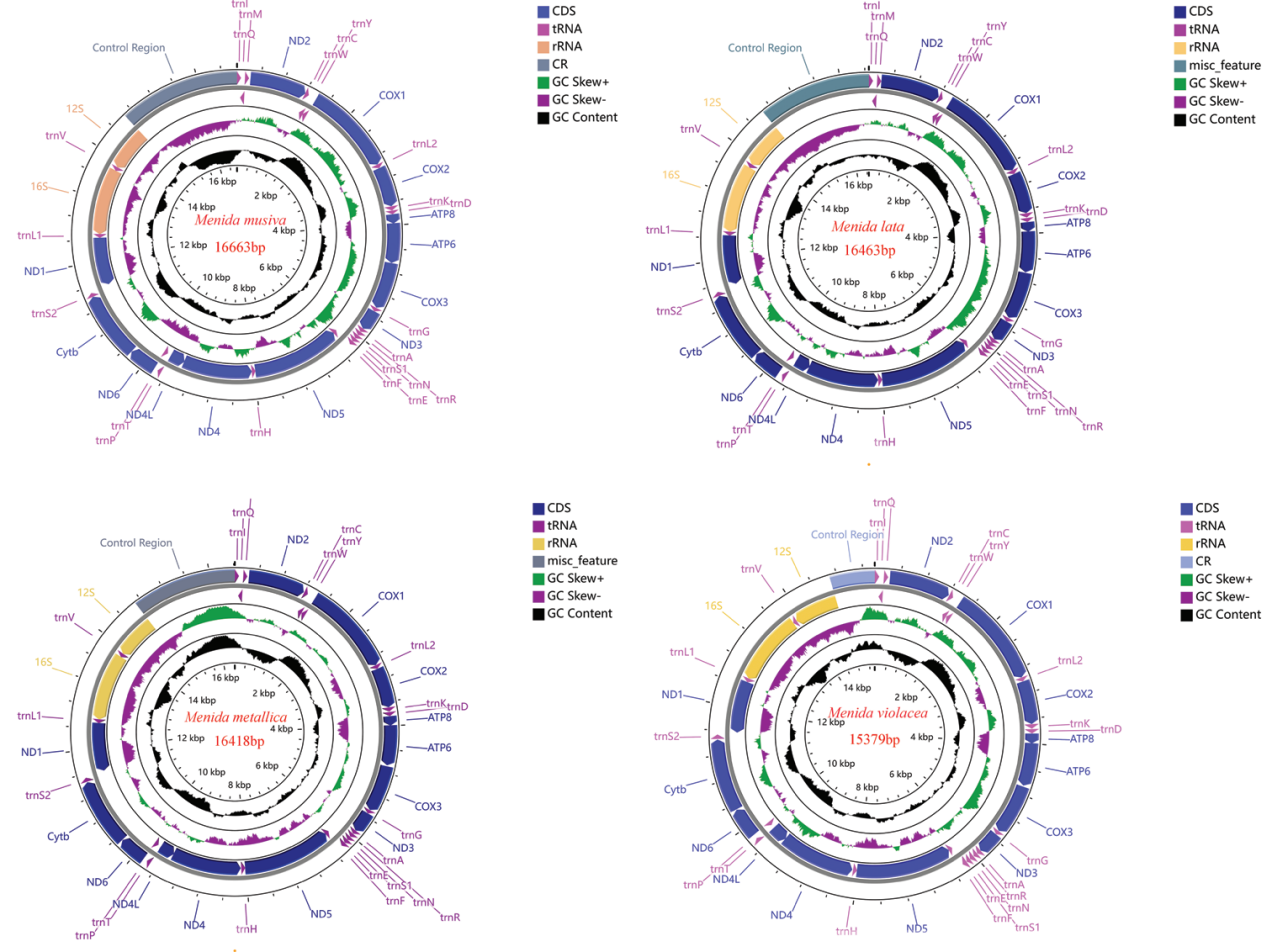
Gene arrangements of the four complete mitochondrial genomes.

We studied the relationship among four species of *Menida* (three newly sequenced species and one species downloaded from NCBI). All four mitogenomes are double-strand circular DNA molecules. The total lengths of the mitogenomes of *M.musiva*, *M.lata*, *M.metallica*, and *M.violacea* are 16,663bp, 16,463bp, 16,418bp, and 15,379bp, respectively. The mitogenomes of the four species each contain 37 genes (13 protein-coding genes (PCGs), 22 tRNA genes, and 2 rRNA genes) and a control region (Fig. 1), with 23 genes located on the J-strand and 14 genes on the N-strand. The sequence of genes was consistent with the original gene arrangement of *Drosophilayakuba* Burla, 1954 (Clary and Wolstenholme 1985; Hua et al. 2008) without rearrangement. Nucleotide composition of the complete mitogenome of *M.musiva*: A 42.51%, T 33.70%, C 14.18%, G 9.60%; nucleotide composition of the complete mitogenome of *M.lata*: A 41.95%, T 32.92%, C 15.08%, G 10.05%; nucleotide composition of the complete mitogenome of *M.metallica*: A 41.39%, T 33.51%, C 13.77%, G 11.33%; nucleotide composition of the complete mitogenome of *M.violacea*: A: 42.19%, T: 33.32%, C: 13.86%, G: 11.33%. The four species show similar nucleotide composition (Suppl. material 1: table S1). All the mitogenomes exhibit a strong base composition bias toward AT, ranging from 74.86% to 76.22% in the four species (mean value = 75.37%). Moreover, all mitogenomes have a slightly positive AT-skew (ranging from 0.11 to 0.12, mean = 0.11) and a negative GC-skew (ranging from 0.20 to −0.10, mean = −0.16) (Suppl. material 1: table S1).

The four mitogenomes have similar overlapping regions and gene spacers. The longest intergenic region (31–34bp) of the four species of the genus *Menida* appeared between *trnS2* and *nad1*, and there were mainly three conserved overlaps, with a 8 bp overlap between *trnC*/*trnW* (AAGCTTTA) and a 7 bp overlap between *atp8*/*atp6* and *nad4*/*nad4L* (ATGATAA) (Suppl. material 1: table S2).

### ﻿Protein-coding genes

For the four studied species, nine PCGs (*nad2*, *cox1*, *cox2*, *atp8*, *atp6*, *cox3*, *nad3*, *nad6*, and *cytb*) were found to be coded on the majority strand (J-strand) and four PCGs (*nad5*, *nad4*, *nad4L*, and *nad1*), on the minority strand (N-strand). The longest PCG is *nad5* (1707–1710 bp), while the shortest is *atp8* (159 bp). Five PCGs (*cox1*, *cox2*, *atp8*, *atp6*, and *nad3*) did not vary in length among the four species. Most of the PCGs use ATN (ATT/ATA/ATG/ATC) as initiation codon. TTG was the second most used initiation codon, and was found in *cox1*, *atp8*, *nad1*, and *nad6* (except in *M.musiva*). The coding region of most PCGs ends with the complete termination codon TAA, except *cox1*, *cox2*, and *nad3*, which ended with the incomplete stop codon T (Suppl. material 1: table S2).

**Figure 2.**
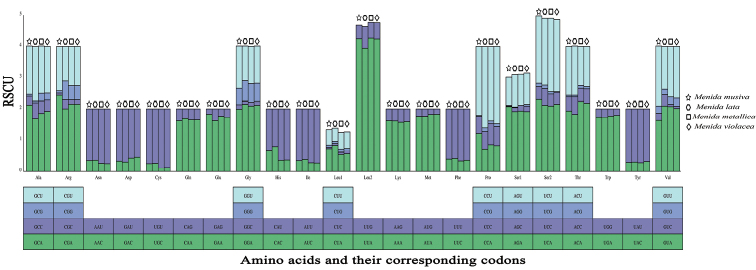
Relative synonymous codon usage (RSCU) in the mitogenomes of four *Menida* species.

Statistics on the relative synonymous codon usage (RSCU) of the four species showed distinct bias and similar codon usage patterns. The most frequently used codons are UUA (Leu2), while the least commonly used codons are AAC (Asn), GAC (Asp), UGC (Cys), CAC (His), AUC (Ile), UUC (Phe), and UAC (Tyr) (Fig. 2). These results indicate that the codons of the mitochondrial protein-coding genes of *Menida* prefer the codon ending with A/T.

**Figure 3.**
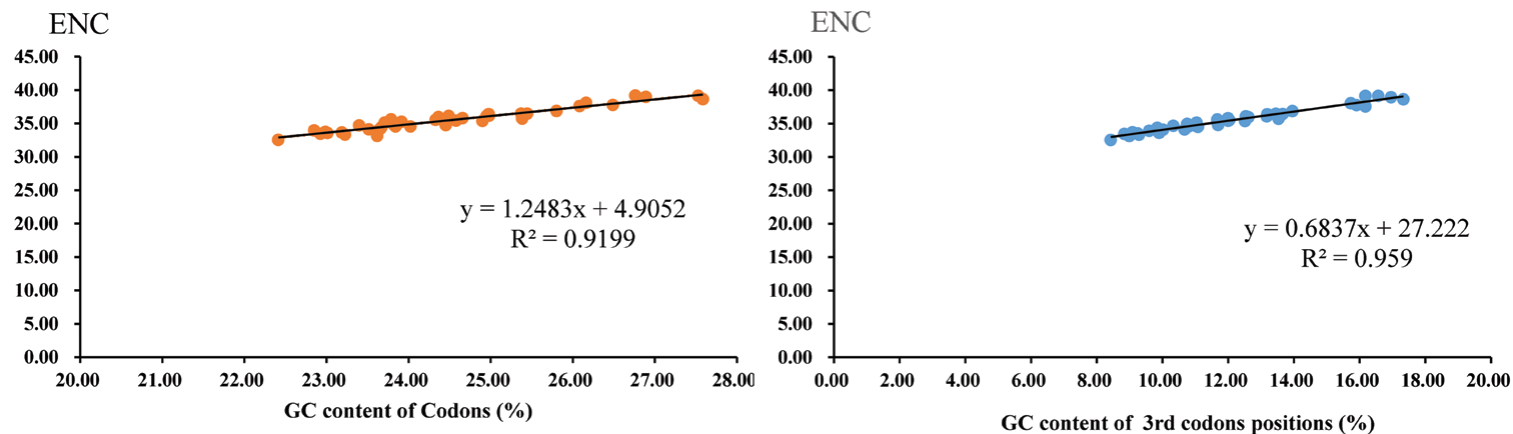
Evaluation of codon bias in the mitochondrial genomes of 40 Pentatominae species.

To further investigate the codon usage bias among Pentatominae species, we analyzed the correlations between ENC (effective number of codons), the GC content of all codons, and the GC content of the third codon positions. We found a positive correlation between ENC and GC content for all codons (R^2^ = 0.9199) and the third codon positions (R^2^ = 0.959) (Fig. 3). These results are consistent with prevailing neutral mutational theories, in which genomic GC content is the most significant factor in determining codon bias among organisms.

**Figure 4.**
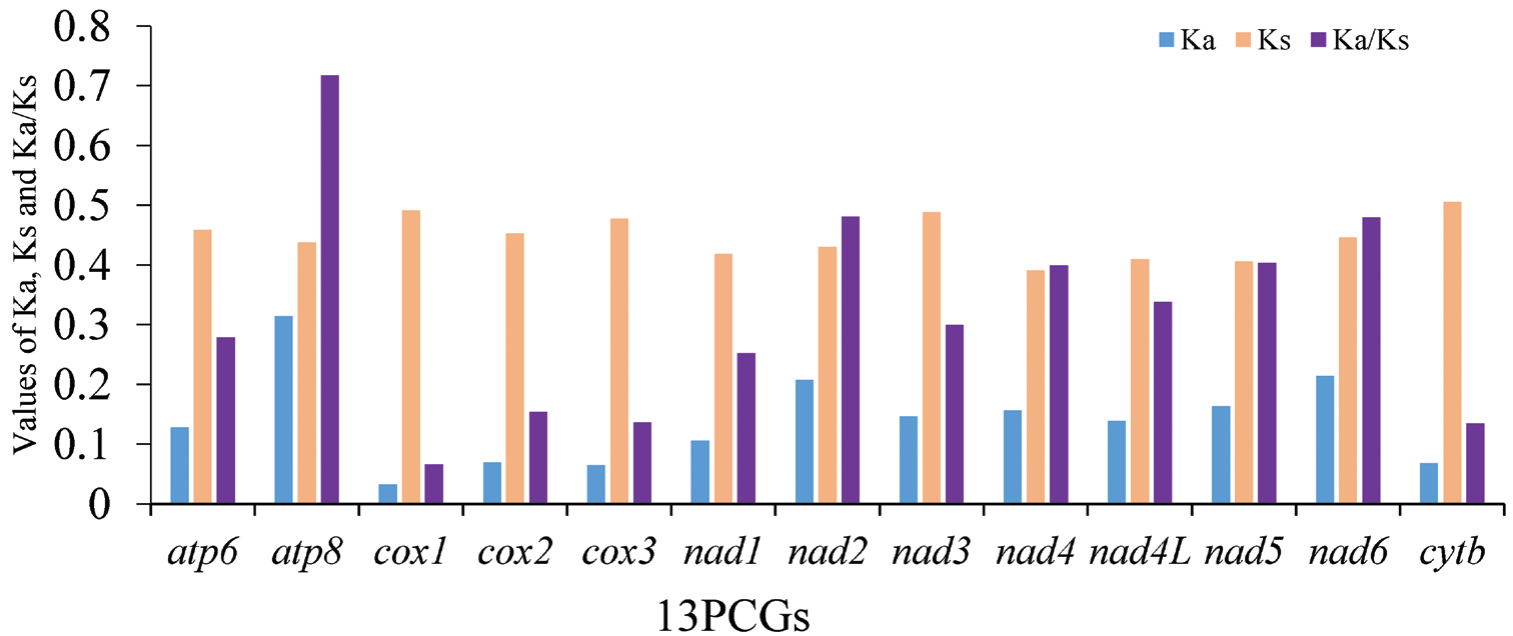
The Ka, Ks, and Ka/Ks values of protein-coding genes within Pentatominae.

The values of Ka (the number of non-synonymous substitutions per nonsynonymous site), Ks (the number of synonymous substitutions per synonymous site), and Ka/Ks were calculated for each PCG, respectively (Fig. 3). The Ka/Ks ratio for all 13 PCGs were below 1.0, indicating evolution under purifying selection. The Ka/Ks ratio of *atp8* was the highest, while that of *cox1* was the lowest. We also observed lower Ka/Ks ratios in the genes that are usually used as a barcode, such as *cox2*, *cox3*, and *cytb*; it is showed that at the nucleotide and amino acid levels, these four genes had the lowest evolutionary rates (Fig. 4).

### ﻿Transfer and ribosomal RNAs

**Figure 5.**
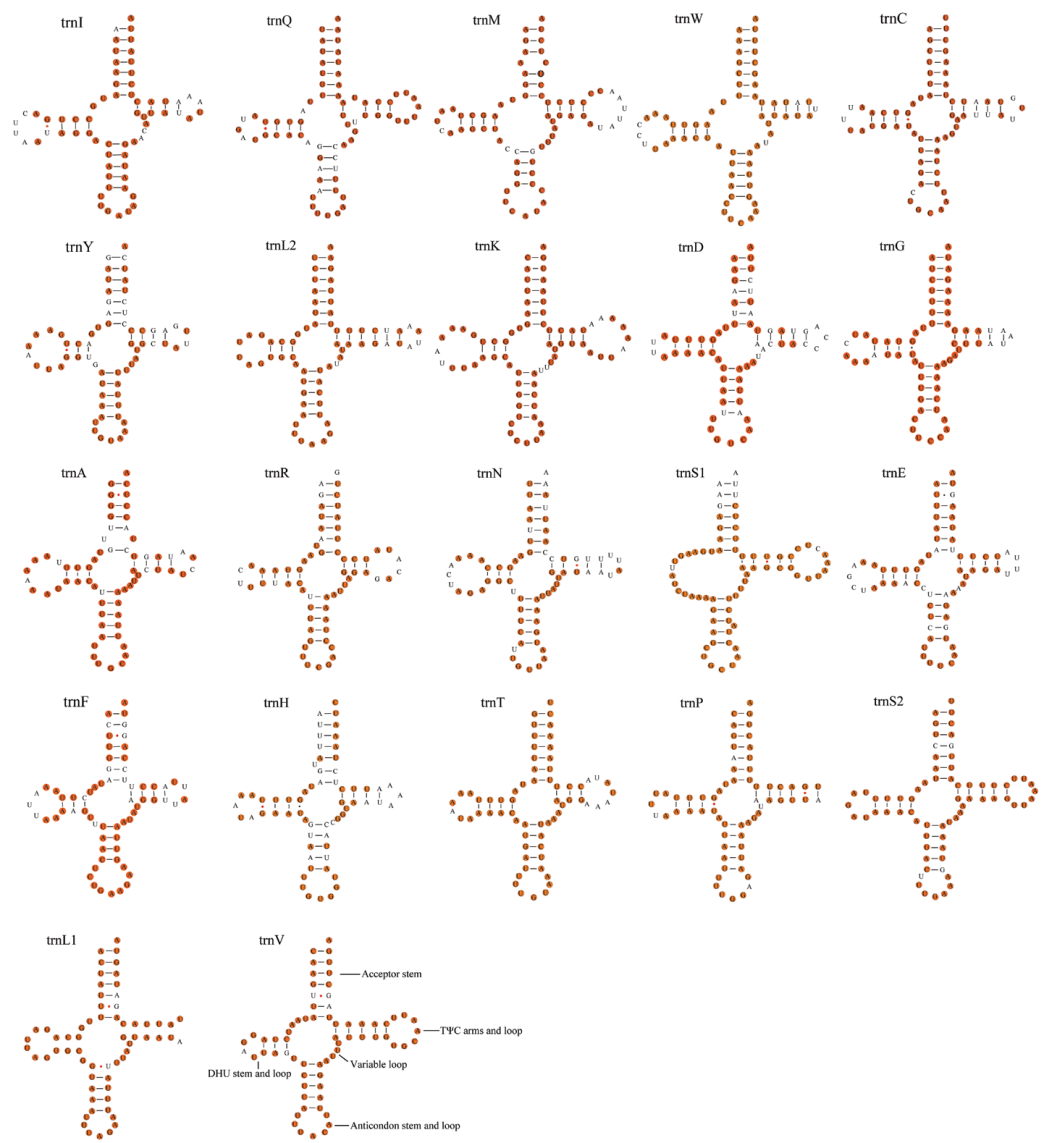
Potential secondary structure of tRNA in *Menidamusiva*. The conserved sites within *Menida* were marked in orange.

The total lengths of the 22 tRNAs of the four species range between 1464 bp (*M.musiva*) and 1484 bp (*M.metallica*), and the length of 22 tRNA genes ranged from 63 to 72 bp. Fourteen tRNA genes (*trnI*, *trnM*, *trnW*, *trnL2*, *trnK*, *trnD*, *trnG*, *trnA*, *trnR*, *trnN*, *trnS1*, *trnE*, *trnT*, *trnS2*) are coded on the J-strand and eight (*trnQ*, *trnC*, *trnY*, *trnF*, *trnH*, *trnP*, *trnL1*, *trnV*) on the N-strand. We found that only *trn S1* lacked the dihydrouridine (DHU) arm, and the remaining 21 tRNA genes can form a typical cloverleaf structure in the four species. All tRNAs in the four mitogenomes use the standard anticodon. Among all the tRNAs of the four species in *Menida*, *trnH* has the weakest conservatism compared with other genes. In addition, 16 wobble G-U pairs were found in 22 tRNAs of *Menida* (Fig. 5), which usually need three-dimensional structure to stabilize.

**Figure 6.**
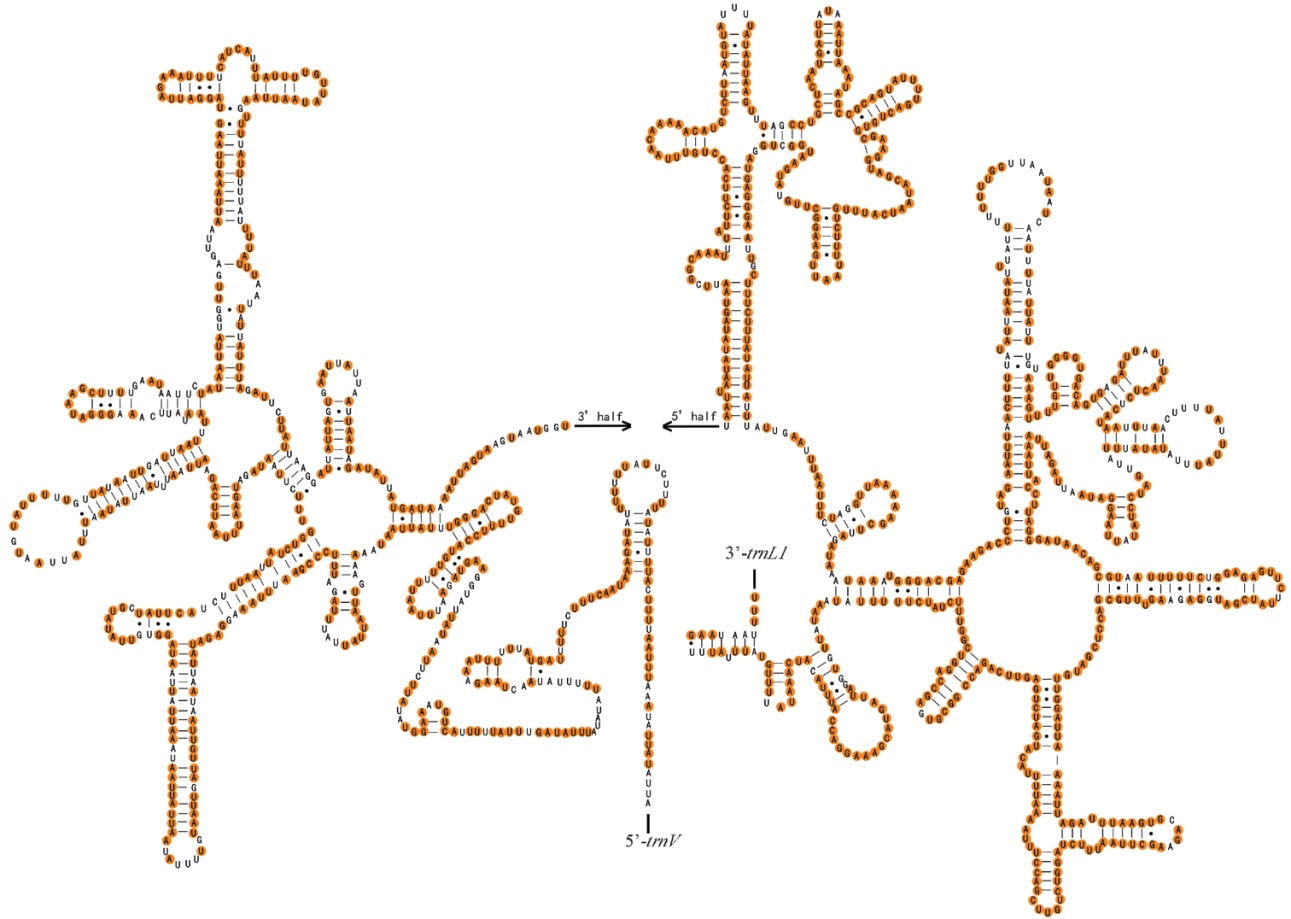
Potential secondary structure of *16S* rRNA in *Menidamusiva*. The conserved sites within *Menida* were marked in orange.

**Figure 7.**
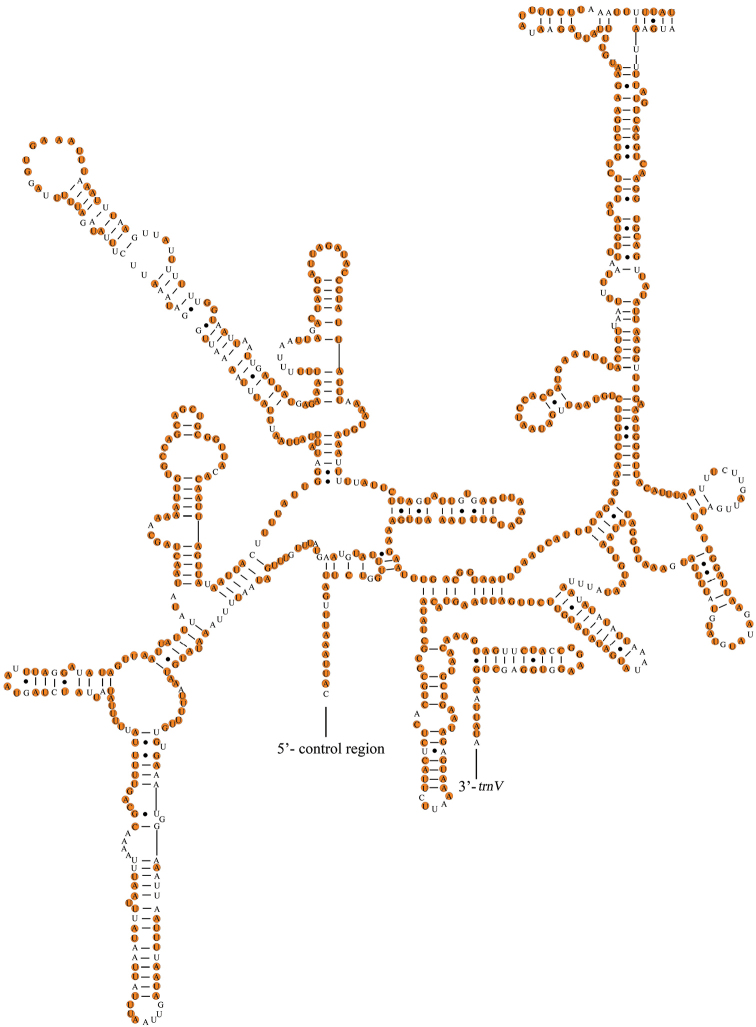
Potential secondary structure of *12S* rRNA in *Menidamusiva*. The conserved sites within *Menida* were marked in orange.

The two rRNA genes (*12S* rRNA and *16S* rRNA) are encoded on the N-strand in these species. The *16S* rRNA gene, ranging from 1277 to 1285 bp in size, is located at a conserved position between *trnL1* and *trnV*. The *12S* rRNA (795–804 bp) was found between *trnV* and the control region. The complete secondary structures of the *12S* rRNA and *16S* rRNA genes are shown in Figs 6, 7, respectively. In *Menida*, *16S* rRNA contained 78.49% conserved sites and *12S* rRNA contained 78.17% conserved sites.

### ﻿Control region

**Figure 8.**
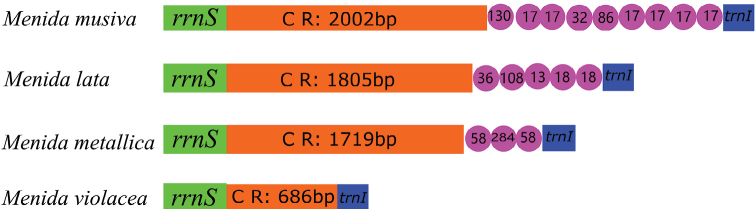
Organization of the control region in the four mitochondrial genomes. The tandem repeats are showed by the magenta circle with repeat length inside. The orange boxes indicate the length of the sequence of the control region.

The control regions located between *12S* rRNA and *trnI* of the four species showed more variation in length, and the length ranged from 686 to 2,002 bp. This variation leads to the difference in the total length of its mitochondrial genome. The AT content in the control area of *M.musiva* (82.82%) was significantly higher than that of the other three species. The longest repeating unit length (284 bp) was found in *M.metallica*. However, no tandem repeats were detected in *M.violacea* (Fig. 8).

### ﻿Phylogenetic relationships

**Figure 9.**
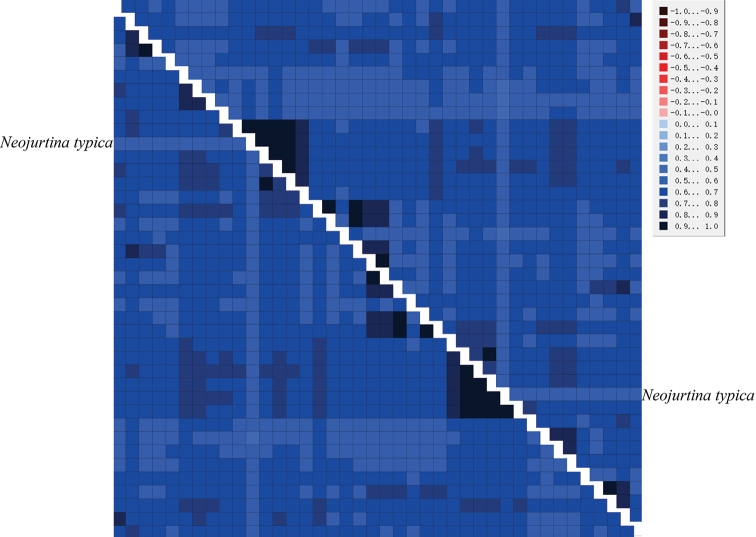
Analysis of heterogeneity of sequence divergence for two datasets (PRT and PR). The heterogeneity of the corresponding sequence relative to other sequences increases as the indicated color becomes lighter. The species with relatively higher sequence heterogeneity are shown.

Before constructing the phylogenetic tree, we performed saturation and heterogeneity analysis on two data sets. Saturation analysis showed that the sequence was not saturated (*Iss* < *Iss. c*, and *p* < 0.05) (Suppl. material 1: fig. S1). Heterogeneity analysis of the two data sets shows that the composition of the sequence has low heterogeneity (Fig. 9). These two data sets are suitable for further phylogenetic studies.

**Figure 10.**
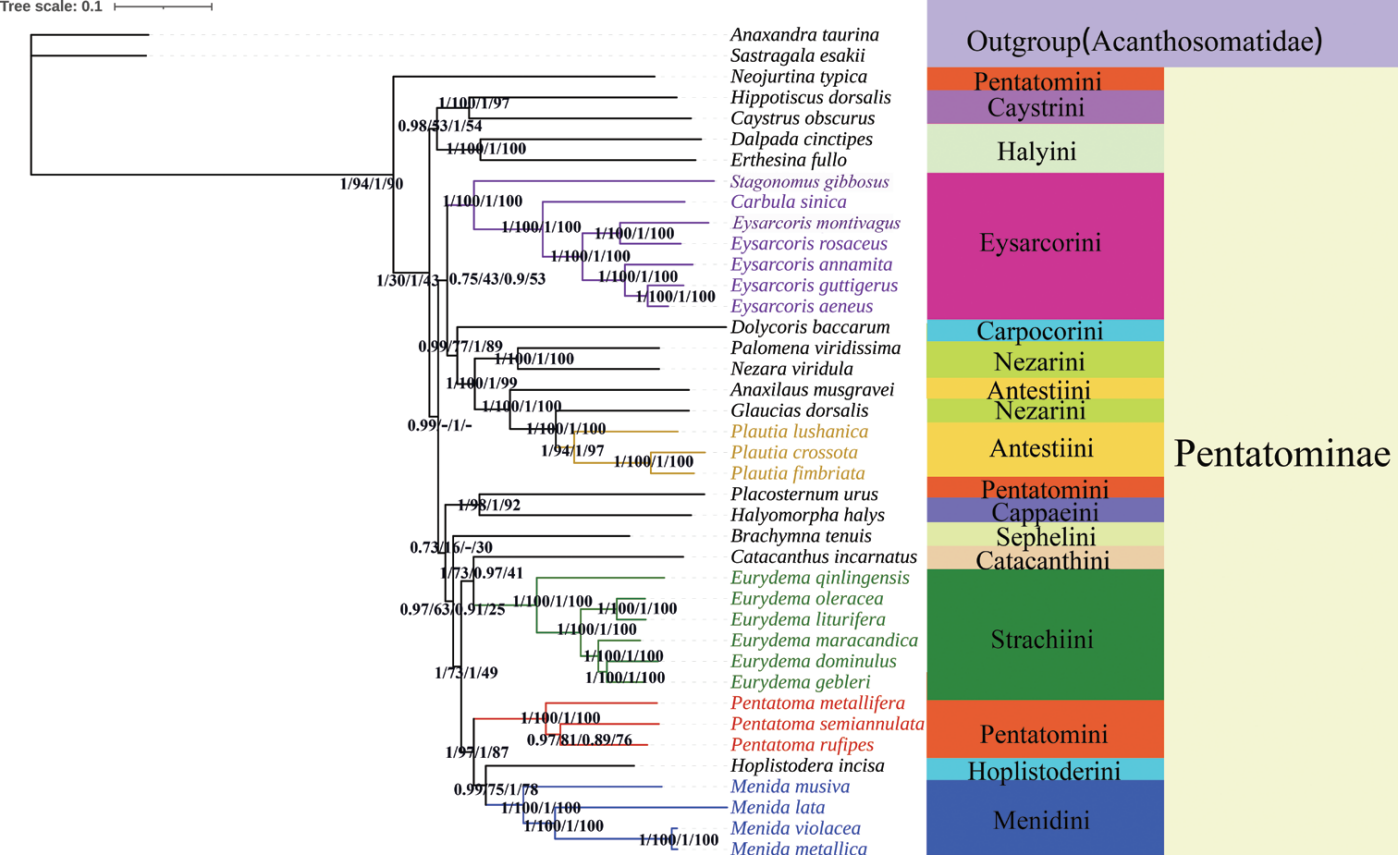
Phylogenetic relationships inferred by the BI and ML method based on the PRT and PR datasets. Numbers on nodes are the posterior probabilities (PP).

We constructed phylogenetic trees of Pentatominae based on the two data sets using BI and ML (Fig. 10). The topological structure of the four trees was highly consistent, and most clades had high posterior probabilities. The phylogenetic positions of the Pentatominae are as follows: (*Neojurtina* + ((Caystrini + Halyini) + (Eysarcorini + (Carpocorini + ((*Palomena* + *Nezara*) + (*Anaxilaus* + (*Glaucias* + *Plautia*))))) + ((*Placosternum* + Cappaeini) + (Sephelini + ((Catacanthini + Strachiini) + (*Pentatoma* + (Hoplistoderini + Menidini))))))). The species *Neojurtinatypica* Distant, 1921 was the earliest diverged lineage within Pentatominae. Other species of Pentatominae were scattered in the phylogenetic tree. *Placosternum* and Cappaeini form a sister-group relationship, and the phylogenetic tree also strongly supports the monophyly of *Pentatoma*. Caystrini and Halyini form a sister group relationship. At the same time, our phylogenetic relationship also shows that the genus *Menida* and Hoplistoderini are closely related within Pentatominae. The four *Menida* species are well grouped; *M.metallica* and *M.violacea* are closely related, and *M.lata* has the longest differentiation time compared to the other species.

## ﻿Discussion and conclusions

In this study, we sequenced the complete mitochondrial genomes of *M.musiva*, *M.lata*, and *M.metallica* based on high-throughput sequencing. Compared with other species of *Menida* with published genomes, no gene rearrangement occurred in the four mitochondrial genomes, and the gene arrangements are conserved, which are consistent with other published mitochondrial genomes of Hemiptera (Lee et al. 2009; Li et al. 2013; Zhang et al. 2013; Wang et al. 2018; Zhao et al. 2018). The size of the complete mitochondrial genome sequence of *Menida* varies greatly, ranging from 15,379 bp in *M.violacea* to 16,663 bp in *M.musiva* (Suppl. material 1: table S2), mainly due to the significant size change of the control region. Previous studies have reported different sizes and different tandem repeats in other Pentatomidae species (Yuan et al. 2015; Zhao et al. 2020; Li et al. 2021). The nucleotide composition of *Menida* is extremely unbalanced (A > T>C > G), showing a strong AT preference. In addition, our analysis of relative synonymous codon usage showed that the codon of protein-coding genes preferred to end with A/T, which was common in all sequenced Pentatomidae (Yuan and Guo 2016). This preference for nucleotide composition is generally thought to be caused by mutational pressures and natural selection (Hassanin et al. 2005).

Most PCGs of mitochondrial genomes of *Menida* use ATN as the initiation codon. TTG is another commonly used start codon and is commonly found in the protein-coding genes (*cox1*, *atp8*, *nad1*, and *nad6*), which is similar to most mitochondrial genomes of Pentatomidae. We found that the stop codon of most PCGs ends with TAA or TAG, while *cox1* and *cox2* end with incomplete stop codon T, which is more conservative in Pentatomidae (Yuan et al. 2015; Zhao et al. 2019b). In addition, most species of Hemiptera also show these three kinds of overlaps, mainly including *trnC*/*trnW* overlap of 8 bp (AAGCTTTA), *atp8*/*atp6* and *nad4*/*nad4l* overlap of 7 bp (ATGATAA) (Zhang et al. 2019).

In the genus *Menida*, tRNAs (except *trnS1*) have a typical shamrock secondary structure and are highly conserved. *TrnS1* lacks DHU arms and only has a ring structure, which is common in many other insect groups. In addition to typical Watson-Crick pairings (G-C and A-U), there are also some atypical pairings such as G-U pairings, and these non-Watson-Crick pairings can be transformed into fully functional proteins by post-transcriptional mechanisms (Chao et al. 2008; Pons et al. 2014).

We obtained highly similar topology based on two different methods of two datasets. Our results are basically consistent with the traditional morphological classification and recent molecular studies (Rider et al. 2018; Chen et al. 2021; Genevcius et al. 2021). Eysarcorini and Strachiini are highly supported as monophyletic (1/100/1/100). We provide support for Roca-Cusachs and Jung’s (2019) suggestion to transfer *E.gibbosus* Jakovlev, 1904 to the genus *Stagonomus* Gorski, 1852. In previous studies, Zhao et al. (2019b) showed that species of *Eurydema* Laporte, 1833 form a sister group with *N.viridula* (Linnaeus, 1758). However, in our study, Catacanthini and Strachiini formed a sister group relationship, and this is also different from the results of Li et al. (2021); more species may be required to support this relationship. Rider et al. (2018) temporarily placed *Plautia* (Stål, 1865) in Antestiini, and our phylogenetic results supported this morphology-based view. Both Antestiini and Nezarini are found non-monophyletic, but combined they form a monophyletic group. At the same time, our phylogenetic analysis also strongly supports the monophyly of the examined species of the genus *Menida*, and the internal relationship of the genus *Menida*: (*M.musiva* + (*M.lata* + (*M.violacea* + *M.metallica*))). However, because there are too few species in this study, the monophyly of the genus *Menida* cannot be well determined, and it is expected to be supplemented by subsequent studies. In addition, in view of the richness of species, it is necessary to analyze more groups, and then clarify the taxonomic status of subfamilies or tribes in Pentatomidae by combining morphological and molecular data.

In the present study, three mitochondrial genomes from the Pentatomidae were analyzed, and the monophyly of some genus has been supported. Due to the richness and diversity of the genus *Menida*, some species within the genus have great morphological variation, so it will be difficult to morphologically identify these species. The addition of these three mitochondrial sequences can provide some data support for the identification of *Menida* species. However, more insect mitochondrial genomes need to be sequenced, which is of great significance for understanding the evolution of mitochondrial genomes and for clarifying the phylogenetic relationship of Pentatomidae.

## Supplementary materials

Supplementary material 1 Supplementary information Data type Phylogenetic (2 files in zip archive). Explanation note In order to explore the genetic diversity and phylogenetic relationship of *Menida* and reveal the molecular evolution of Pentatominae, three complete mitochondrial genomes of *Menida* were sequenced, and the phylogenetic relationships of tribes within the subfamily Pentatominae were studied based on mitochondrial genomes. The mitochondrial genomes of three species (*Menidamusiva*, *M.lata* and *M.metallica*) were 16,663bp, 16,463bp and 16,418 bp, respectively, encoded 37 genes, including 13 protein-coding genes (PCGs), two rRNA genes, 22 tRNA genes and a control region. We compared and analyzed the mitochondrial genomes characteristics of *Menida*, and constructed the phylogenetic tree of Pentatominae based on the mitochondrial genomes datasets by Bayesian method. The results showed that gene arrangements, nucleotide composition, codon preference, gene overlaps and RNA secondary structures were highly conserved within the *Menida*, and had more similar characteristics in Pentatominae. Phylogenetic analysis showed highly consistent topological structures based on BI methods, which strongly supported that the genus *Menida* belongs to the Pentatominae and is the earliest branch of the sequenced pentatominae species. In addition, (Pentatomini+Strachiini) and (Nezarini+Antestiini) were found to be stable sister groups in the evolutionary branch of Pentatominae. The results of this study enrich the mitochondrial genomes databases of Pentatominae, and have important significance for further elucidate the phylogenetic relationship of Pentatominae. https://binary.pensoft.net/file/791070This dataset is made available under the Open Database License (http://opendatacommons.org/licenses/odbl/1.0/). The Open Database License (ODbL) is a license agreement intended to allow users to freely share, modify, and use this Dataset while maintaining this same freedom for others, provided that the original source and author(s) are credited.Xiaofei Ding
